# Bacterial vaginosis and antibacterial susceptibility pattern of asymptomatic urinary tract infection in pregnant women at a tertiary care hospital, Visakhaptn, India

**Published:** 2019-12

**Authors:** Appikatla Madhu Bhavana, Pilli Hema Prakash Kumari, Nitin Mohan, Vijayalakshmai Chandrasekhar, Payala Vijayalakshmi, Rongala Venkata Manasa

**Affiliations:** 1 Department of Microbiology, GITAM Institute of Medical Sciences and Research, Visakhapatnam, Andhra Pradesh, India; 2 Department of Obstetrics and Gynaecology, GITAM Institute of Medical Sciences and Research, Visakhapatnam, Andhra Pradesh, India; 3 Department of Community Medicine, GITAM Institute of Medical Sciences and Research, Visakhapatnam, Andhra Pradesh, India

**Keywords:** Antibiotic susceptibility pattern, Bacterial vaginosis, Pregnant women, Urinary tract infection

## Abstract

**Background and Objectives:**

The association between bacterial vaginosis and urinary tract infection (UTI) in pregnant women is at a greater risk comparatively than patients with bacterial vaginosis or UTI. Bacterial vaginosis and asymptomatic UTI both pose risk for mother and fetus. Early diagnosis and treatment can save the life of both. The present investigation was aimed to find out the magnitude of asymptomatic bacteriuria in pregnant women with noticeable bacterial vaginitis attending antenatal outpatient and inpatient of a tertiary care hospital and to identify the organisms causing it.

**Materials and Methods:**

A total of 117 antenatal women from different age and parity groups with different gestational ages were included in the study. The samples were subjected to standard microbiological techniques for identification of microorganisms. While performing Per speculum examination, vaginal secretions were collected from the posterior fornix. Swabs from the posterior fornix were tested for pH using litmus paper. A wet mount and Gram smear was made and examined for the presence of bacteria, polymorphs and clue cells indicating bacterial vaginosis. Amsel’s criteria and Nugent scoring system were applied for diagnosis of bacterial vaginosis. Antibiotic susceptibility of the isolated bacteria was performed using Kirby-Bauer method.

**Results:**

Bacterial vaginosis infection rate (62.3%) was common in the present study followed by asymptomatic UTI (n=60, 51%). It was also observed that asymptomatic urinary tract infection (UTI) with Bacterial vaginosis prevalent rate was 49 (41.8%) in the current study.

**Conclusion:**

Bacterial vaginosis was more common than asymptomatic bacteriuria in pregnant women. It is recommended that antenatal health care facilities should incorporate screening of vaginitis among pregnant women to prevent the complications of pregnancy. And those women with Bacterial vaginosis should be screened for UTI. Proper use of antibiotics should be encouraged, abuse of antibiotics should be in check.

## INTRODUCTION

Vaginal infection with bacterial vaginosis and candidiasis is a worldwide health problem for pregnant women. Vaginitis results from inflammation and infection of vagina with assorted spectrums of pathogenic microorganisms, identified in the vaginal normal flora (1). Even though there are a number of patients with vaginal infections their presentation may be symptomatic or asymptomatic (2). Bacterial vaginosis (BV) is recognized by increase in vaginal pH, homogenous white discharge in which normal vaginal flora (Lactobacilli) is substituted by a mixed microbial population of aerobic, microaerophilic organisms and even some anaerobic microorganisms like *Gardnerella vaginalis, Mycoplasma hominis, Mobiluncus* spp. colonize vagina predominantly and cause bacterial vaginosis (3). Nearly 10–30% women experience bacterial vaginosis in pregnancy. It leads to pre-mature membrane rupture, pre-term labor and miscarriages. Vulvovaginal candidiasis (VVC) is due to over growth of yeast specifically *Candida* species, in the vaginal mucosal area. Infection with *Candida albicans* is common in most of the diagnosed VVC cases nearly ranging from 80–90%, and other species like *C. glabrataor C. tropicalis* are reported less commonly. Using adequate pharmacotherapy (anti-fungal and antiseptic agents) and evasion of contributing factors like douching, wearing tight pants etc., VVC can be resolved within a short period of time (4). The higher estrogen levels and higher glycogen content in vaginal secretions in pregnancy may be responsible for woman’s higher risk of VVC. Since VVC is so frequent in women during pregnancy, it is significant to identify the pathology of the disease in addition to the safety or risks of drugs that are used during pregnancy to treat it (5).

Urinary tract infection (UTI) is also one of the major factors of pre-term labor in infected pregnant women. In UTI, pathogenic organisms are identified in the urine, urethra, bladder, kidney. Occasionally, the microbial count may also be due to specimen contamination, specifically when multiple species of bacteria are isolated. In symptomatic patients, a smaller number of bacterial counts (10^2^ to 10^4^ /mL) may indicate infection (6). Identification of asymptomatic bacteriuria (ASB) in pregnancy is the key step because it subsequently may lead to symptomatic infection and a variety of pregnancy related complications like anemia, hypertension, phlebitis, low birth weight babies, preterm labor, abortions (7). Asymptomatic bacteriuria (ASB) has been reported among 13.0% pregnant women. From the literature study, it was found that very few studies have been reported from India, as ASB screening is not carried out regularly may be due to cost propositions. The increased risk factor for UTI in women may be due to short urethra, absence of prostatic secretions, pregnancy and easy contamination of urinary tract with faecal flora. The physiological increase in plasma volume during pregnancy decreases urine concentration and up to 70% of pregnant women develop glycosuria, which is considered to encourage bacterial growth in the urine (8). UTIs during 3^rd^ trimester increase the relative risk for mental retardation or developmental delay, as well as fetal death. The association between BV and UTI in pregnant women was first reported in the year 1989 by Hooten et al. (9) who found that women suffering from BV are at a greater risk of UTI comparatively than others (10). It is risk for both mother and fetus and a single step of early diagnosis and treatment can save the life of both. Hence, the present investigation was aimed to study the efficacy of Nugent’s score and Amsel’s criteria in the detection of bacterial vaginosis and to find out the magnitude of asymptomatic bacteriuria in pregnant women with noticeable bacterial vaginitis attending antenatal outpatient and inpatient of a tertiary care hospital.

## MATERIALS AND METHODS

The study was conducted at the Department of Microbiology and Gynaecology of the GIMSR (GITAM Institute of Medical Science and Research), Visakhapatnam. It is a tertiary level hospital and the study was performed on total 117 numbers of samples. The study was conducted following approval of Institutional Ethics Committee and the study subjects gave the informed consent form. A convenient sampling technique was used for the data collection from the study population, who satisfied inclusion criteria to participate in the study.

### Inclusion criteria.

Pregnant women of 10 weeks gestation to term attending with complaints of vaginal discharge attending the antenatal clinic.

### Exclusion criteria.

Non pregnant women and pregnant women with symptomatic UTI.

Patients between 10 weeks to 40 weeks of gestation with complaints of vaginal discharge were included in the study. Any preganat women with symptoms of urinary tract infection were excluded from the study. Vaginal samples were collected in the OPD. The specimens collected were analyzed using Amsel and Nugent’s scoring methods and urine samples were collected from all the patients for culture.

### Collection of vaginal sample, wet mount.

**Fig. 1.**
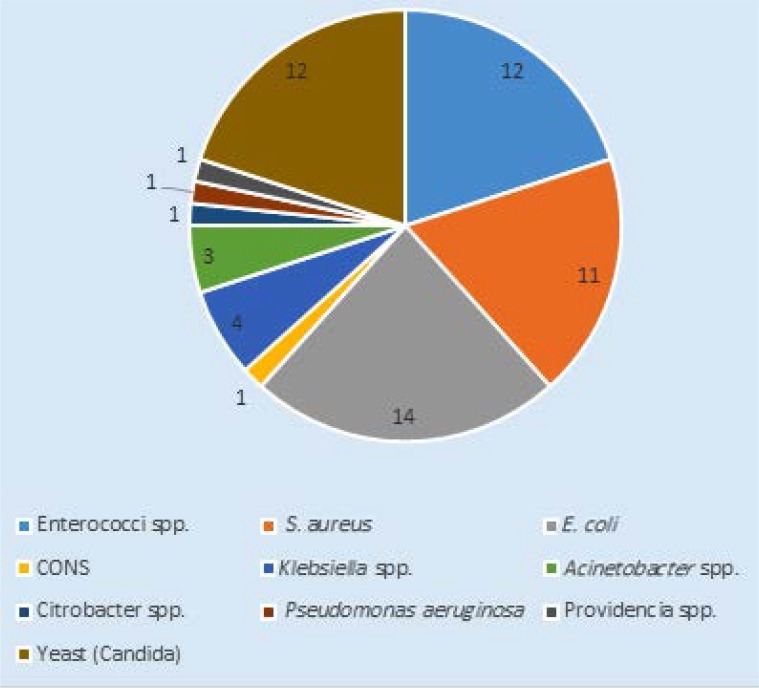
Incidence of pathogenic microorganisms causing UTI

After assurance of patient, Per speculum examination was done and the vaginal secretions from the posterior fornix were collected. First swab from the posterior fornix will be tested for pH using litmus paper. A wet mount and Gram stain smear was made and sent to Microbiological laboratory for examination. The wet mount of vaginal discharge was examined for the presence of bacteria, white blood cells and unusual cells called clue cells. Detection of clue cells indicates bacterial vaginosis (Fig. 1). Amsel’s criteria and Nugent scoring system are among the most commonly used diagnostic methods. Nugent scoring system was considered the gold standard and sensitivity, specificity, positive predictive value and negative predictive value of Amsel’s criteria were compared with those of Nugent scoring system.

Nugent scoring system, developed by Nugent et al. (12), is based on Gram staining and observing the number of lactobacilli and other morphotypes (different shapes of *Gardenerella vaginalis, Prevotella* species, and *Mobiluncus* species) which are scored between 0 and 10, where scores 7–10 indicates bacterial vagonosis. Its high sensitivity has led to its recognition as the gold standard of BV.

The presence of a homogeneous vaginal discharge, pH> 4.5 of vagina, the incidence of any clue cells in the wet mount of the vaginal discharge and a positive whiff test constitutes Amsel’s composite criteria. According to Amsel, if 3 of the 4 criteria are positive, the patient has bacterial vaginosis (11). The pH of the vaginal secretions can be obtained by placing a sample from the lateral wall of the vagina on pH paper. The paper should include a range of pH from 4 .0 to above 5.0. The normal pH is 4.5 or less. The whiff test is a test for the fishy odor that occurs in bacterial vaginosis (previously called Gardnerella vaginitis and nonspecific vaginitis). A drop of KOH is mixed with some vaginal discharge. A positive test is abnormal and consists of a characteristic fishy odor.

Clue cells are the vaginal epithelial cells covered with cocobacilli, as a result their edges which generally have a sharply defined cell border became indistinct or stippled. Presence of clue cells indicates BV. If the clue cells constitute 20% or more of the epithelial cells in the high power field it is considered positive. It should also be noted that, presence of motile trichomonads, budding yeast cells and pseudohyphae (12) in the sample.

### Collection and microbiological examination of urine sample.

A total of 117 antenatal women with history of discharge from both OP and IP (GYN and OBG Departments) of different ageand parity groups with different gestational ages were included in the study. Clean-catch midstream urine was collected from each patient into a sterile universal container. 0.05 ml of uncentrifuged urine sample was microscopically examined at high magnification for presence of pus cells, red blood cells, epithelial cells, casts, crystals, and yeast-like cells (*Candida* species). Observation of 1 leucocyte per 7 high power fields is significant and corresponds to 10^4^ leucocytes per ml. Samples were cultured on dried plates of Blood Agar and Cysteine Lactose Electrolyte Deficient Agar (CLED). Plates were incubated aerobically at 37°C for 24 h of incubation. Bacterial growth of 10^5^ cfu/ml or more of pure isolates were regarded as significant for infection (13). The isolated organisms from culture plates were identified by Gram staining and biochemical reactions. The phenotypic tests were used for identification of isolated organisms (13).

### Antibiotic susceptibility testing.

Antimicrobial susceptibility testing was performed using Kirby Bauer disk diffusion method according to CLSI guidelines using Muller-Hinton agar (MHA) plates. The plates were incubated at 37°C for 16–18 h. The antibiotic discs used in this study were listed in the Tables. The inhibition zone was measuredand compared with the CLSI guidelines (14).

## RESULTS

### Demographic variable: age.

Of 117 pregnant women attended OP and OP (GYN and OBG dept), the maximum number of cases were in the age group 20–29 years i.e. 103 (88%) followed by 30–39 years 9 (7.7%), and <20 years 5 (4.3%) and no cases were reported in the age group 40 and above.

### Gestational age.

The majority of the patients with complaints of discharge fall in 3^rd^ trimester 84 (71.8%) of pregnancy followed by 2^nd^ trimester 26 (23%) and 1^st^ trimester 7 (5.9%).

### Detection of BV based on Amsel’s criteria.

In Amsel’ scriteria, the positivity of 3 factors indicates bacterial vaginosis. Increased homogenous discharge was found in all 117 patients (100%), whiff’s test positive was seen in 60 patients (51.2%), clue cells were observed in 55 patients (47%) and pH greater than 4.5 was seen in 101 patient high vaginal swabs (86.3%).

### Prevalence of bacterial vaginitis based on Nugent’s score and detection of BV based on Amsel’s criteria.

Based on Nugent’s scoring system ranging from 0–10 (15), maximum numbers of cases i.e. 93 were reported in the score 7–10 (79.5%) followed by 20 case in the nugent’s score 0–3 (17.1%) and 4 cases in the score 4–6 (3.4%).

By comparing the results of bacterial vaginosis by Amsel’s criteria and Nugent’s score, it was found that the number of positive cases based on Amsel’s criteria was 83 (70.9%) and negative cases 34 (29.1%) and the number of positive cases based on Nugent’s scoring system was 93 (79.5%) and negative cases 24 (20.5%).

### Detection of vaginitis in different gestational age groups.

Table 1 showed that, 80 cases (68.3%) were reported to have vaginitis out of 117 samples. The prevalence of infectious bacterial vaginitis among pregnant women was found to be high 73 (62.3%) among 117 samples. BV was the most prevalent type of vaginitis followed by VVC (3.41%) and mixed infection BV+VVC3 (2.5%). As many cases were positive in BV than VVC and BV+VVC infection types the statistical values were insignificant. The results also revealed that maximum numbers of vaginitis cases were recorded in the 3rd trimester of pregnancy 56 (67%).

**Table 1.** Gestational age wise distribution of vaginitis among infected women

**Gestational age groups (Trimesters)**	**Number of cases**	**BV**		**VVC**		**BV+VVC**		**Number of Positive cases**	

		**F**	**%**	**F**	**%**	**F**	**%**	**F**	**%**
1	7	5	7	0	0	0	0	5	6
2	25	19	26	1	29	1	33.3	22	28
3	93	49	67	3	71	2	66.7	56	70
Total	117	73 (62.3%)	100	4 (3.41%)	100	3 (2.56%)	100	80 (68.3%)	100

N=117 Chi-square= 0.77 Df=4 P-value=0.94

### Detection of asymptomatic UTI.

Among 117 urine samples subjected to microbiological analysis, 60 microbial isolates were identified. The major isolate was *E. coli* 14 (12%) followed by *Enterococcus* species 12 (10%), *Staphylococcus aureus* 11 (9%), *Klebsiella* species 4 (3%), *Acinetobacter* species 3 (2%). Very few isolates were recorded in the species of *Citrobacter, Pseudomonas, Providencia* and coagulase negative staphylococci (CONS) 1 (1%). The growth of *Candida* species was identified in 12 (10%) samples (Fig. 1).

### Asymptomatic UTI with BV.

Out of 117 patients, the mixed infection bacterial vaginosis and urinary tract infection was found in 49 patients (41.8%).

### Incidence of BV, UTI and their associations.

Bacterial vaginosis infection rate 73 (62.3%) was more common in the present study followed by asymptomatic UTI 60 (51%). It was also observed that asymptomatic UTI with BV rate was 49 (41.8%) in the current study. On the other hand, very few cases reported only vulvovaginits 7 (5.9%) and bacterial vaginosis with vulvovaginits 3 (2.3%). Hence bacterial vagiosis was more in pregnant women than asymptomatic bacteriuria in the present study.

### Antibiotic susceptibility testing of isolated microorganisms.

**Fig. 2.**
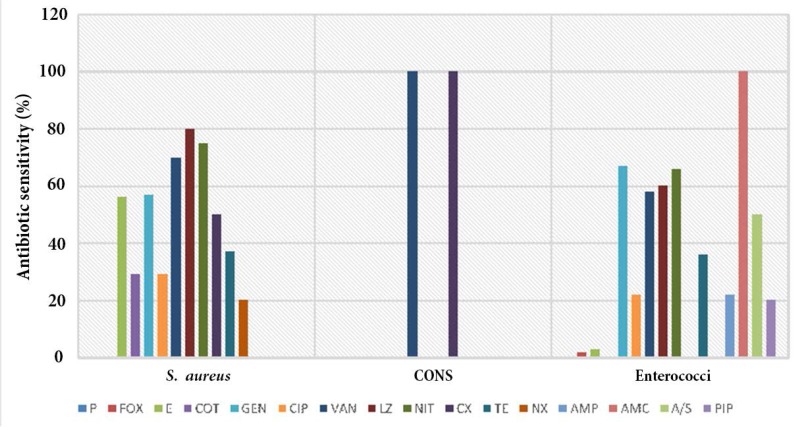
Antibiotic sensitivity pattern of Gram-positive cocciby Kirby-Bauer method **Antibiotics abbreviations:** AMP: ampicillin, AMC: Amoxycillin/clavulanic acid, P: penicillin, Fox: cefoxitin, E: erythromycin, COT: co-trimoxozole, Gen: gentamycin, CIP: ciprofloxacin, VAN: vancomycin, LZ: linezolid, NIT: nitrofurantion, CX: cefixime, TE: Tetracycline, NX: nalidixic acid, CAZ: ceftazidime, CAC: ceftazidime/clavulanic acid, AK: amikacin, IMP: imipenem, CPM: cefipime, PIP: piperacillin, TZP: piperacillin/tazobactam, ETP: ertapenem, MEM: meropenem, LVX: levofloxacin, CFP/S: Cefoperazone/sulbactam, A/S: Ampicillin/sulbactam, CXM: cefuroxime, CTX: cefotaxim

Strains of *S. aureus* showed high susceptinilty to linezolid (80%) followed by nitrofurantoin (75%) and vancomycin (70%) whereas CONS showed maximum sensitivity (100%) to vancomycin and cefixime (Fig. 2). The second most prevalent organism of the study Enterococci reported maximum sensitivity to Amoxycillin/clavulanic acid (100%) followed by Nitrofurantoin (66%) and linezolid (60%) (Fig. 2). The results of antibiotic susceptibility testing of Gram-negative bacteria revealed that, *E. coli* the foremost predominant organism of the study were 71% sensitive to ertapenem (Table 2). Majority of the strains of *Klebsiella* species. was highly sensitive to imipenem (67%) followed by citrobacter species. sensitive highly to piperacillin/tazobactam and imipenem (100%) (Table 2). All the strains of *Providencia* were 100% sensitive to all tested antibiotics except co-trimoxazole. The study also isolated non-fermenting Gram-negative bacilli *A. baumanii* which exhibited maximum sensitivity to ertapenem (66%) and levofloxacin (50%) and *P. aeruginosa* was 100% sensitive to nitrofurantoin (Table 3).

**Table 2.** Antibiotic sensitivity pattern of Gram-negative bacilliby Kirby-Bauer method

**Microorganisms**	**FOX E**	**COT**	**GEN**	**CIP VAN LZ**	**NIT CX TE**	**NX**	**AMP**	**AMC**	**A/S PIP**	**CTX**	**CAZ**	**CAC**	**AK**	**IMP**	**TZP**	**CMP**	**ETP**
*E. coli*	8	25	13	50	33		14	14		0	0	11	31	70	0	0	71
*Klebsiella* spp.	0	0	25	0	25	0			33	0	0	0	33	67		0	0
*Citrobacter* spp.	0			0	0	0	0		0				0	100	100	0	
*Providencia* spp.	100	0	100						100		100	100	100	100	100		

**Table 3.** Antibiotic sensitivity pattern of Non-fermentative Gram-negative bacilliby Kirby-Bauer method

**Microorganisms**	**ETP**	**A/S**	**PIP**	**CFP/S**	**CAZ**	**CAC**	**AK**	**IMP**	**CPM**	**MEM**	**LVX**
*Pseudomonas aeruginosa*			23.5		0	8.3	2.4	4.8	11.8		
*Acinetobacter baumannii*	66	34		34	0	100	0		34	0	50

## DISCUSSION

In the present study, the incidence of infectious vaginitis was found to be high (n=80, 68.3%). Bacterial vaginitis (n=73, 62.3%) was seen more followed by vulvovaginitis (n=4, 3.41%) and BV +VVC (n=3, 2.56%). This rate of bacterial vaginitis reported in the current study was quite high and at the same time the rate of BV+VVC was less than the studies reported earlier. Lamichhane et al. (10) showed 40.0% positive cases of vaginitis in which bacterial vaginitis was more common 39.13% and BV+VVC (30.43%). Similarly Shrestha et al. (16) concluded that BV was more common (52.6%) followed by BV+VVC (16.7%). Afrakhteh (17) showed 62.7% of bacterial vaginitis cases. By observing the demographic variable age, maximum numbers of cases were recorded in the age group 20–29 (88%). These findings were similar to the results of Lamichhane et al. (10), Nithyalakshmi and Vijayalakshmi (18), Sujatha and Manju (19). The higher rate of infection particularly in this age group might be due to it being the most active reproductive age group and high sexual exposure. The study observed more cases in the gestational age of third trimester 84 (71.8%) which coincides to the findings of Lamichhane et al. (10) and Afrakhteh (17). Higher infection rate in third trimester of pregnancy might be due to increased estrogen and corticoid levels that interrupt vaginal acid level and reduce vaginal defense mechanisms against opportunistic infections caused by organisms like *Candida*. The involvement of the lower urinary tract, leading to asymptomatic bacteriuria is the most common cause of UTI during pregnancy. Studies proved that 25%–40% of untreated pregnant women with asymptomatic bacteriuria will eventually develop to acute pyelonephritis as the most common cause of pre-delivery hospitalization. The asymptomatic UTI 60 (51%) was high in pregnant women followed by asymptomatic UTI + BV 49 (41.8%) which coincides to the findings of Amsel et al. (20) and Hill et al. (21). The prevalence rate of bacterial vaginitis in asymptomatic bacteriuria patients is high in the present study while comparing to the studies of Harmanli et al. (22) showed only 22.4% of UTI+BV cases and 9.7% asymptomatic UTI where as Lamichhane et al. (10) reported 23.4% UTI+BV and 10.2% asymptomatic UTI. It seems that sexual intercourse has a significant confounding role in the involvement of UTIs and bacterial vaginosis (23). In women UTI will occur when uropathogens almost always from the fecal flora colonize the vagina, go up into the bladder and in some cases the kidney. Loss of the vaginal lactobacilli may predispose women to acquisition of genitourinary infections. The colonization of different pathogenic microorganism is favored by host behavioral factors such as spermicides use, sexual intercourse and moreover due to increase in vaginal pH as a result of decrease or alteration of normal flora of vagina i.e. lactobacilli to the vagina as a result of BV which ultimately results in UTI (9).

The predominant organisms isolated from BV+ asymptomatic UTI patients were *E. coli* (12%) followed by enterococci (10%), *S. aureus* (9%) and *Klebsiella* spp. (3%). In accordance with our study, Lamichhane et al. (10), Carel (24), Afrakhteh (17) reported *E. coli* was the major isolate. Enterococci was reported to be the second most predominant organism in the current study and very few reports showed enterococci was one of the predominant isolate. Amiri et al. (25) showed only 4% enterococci cases in pregnancy. Majority of the strains of *E. coli* was highly sensitive to antibiotic ertapenem and quinolone compound ciprofloxacin and multi-drug resistant isolates were isolated in the present study which coincides to the reports of Lamichhane et al. (10). The higher rate of MDR isolates may be due to unprudent use of antibiotics which is in particular true for developing countries where antibiotics are prescribed irrationally to the patients by the medical chemists but not by the clinical physicians. The antibiotics generally prescribed to pregnant women for the treatment of UTI include nitrofurantoin, amoxicillin, amoxicillin-clavulanate, cephalexin, cefuroxime, erythromycin, sulfisoxazole and azole compounds for the treatment of vulvovaginitis.

## CONCLUSION

It is recommended that antenatal health care facilities should incorporate screening of vaginitis among pregnant women to prevent the complications of pregnancy and those women with BV should be screened for UTI. Proper use of antibiotics should be encouraged and abuse of antibiotics should be in check. Since douching, sexual intercourse are risk factors of BV, preventive measures should be undertaken.
